# Growth and optical properties of ZnO nanorod arrays on Al-doped ZnO transparent conductive film

**DOI:** 10.1186/1556-276X-8-158

**Published:** 2013-04-08

**Authors:** Suanzhi Lin, Hailong Hu, Weifeng Zheng, Yan Qu, Fachun Lai

**Affiliations:** 1 College of Physics and Energy, Fujian Normal University, Fuzhou 350108, People’s Republic of China; 2 Analytical and Testing Center, Southwest University of Science and Technology, Mianyang 621010, People’s Republic of China

**Keywords:** ZnO nanorod, Al-doped ZnO films, Catalyst-free growth, Optical properties, 81.07.-b, 61.46.Km, 78.67.-n

## Abstract

ZnO nanorod arrays (NRAs) on transparent conductive oxide (TCO) films have been grown by a solution-free, catalyst-free, vapor-phase synthesis method at 600°C. TCO films, Al-doped ZnO films, were deposited on quartz substrates by magnetron sputtering. In order to study the effect of the growth duration on the morphological and optical properties of NRAs, the growth duration was changed from 3 to 12 min. The results show that the electrical performance of the TCO films does not degrade after the growth of NRAs and the nanorods are highly crystalline. As the growth duration increases from 3 to 8 min, the diffuse transmittance of the samples decreases, while the total transmittance and UV emission enhance. Two possible nanorod self-attraction models were proposed to interpret the phenomena in the sample with 9-min growth duration. The sample with 8-min growth duration has the highest total transmittance of 87.0%, proper density about 75 μm^−2^, diameter about 26 nm, and length about 500 nm, indicating that it can be used in hybrid solar cells.

## Background

ZnO, one of the most important metal oxides, has a wide bandgap of 3.37 eV and a high exciton binding energy of 60 meV at room temperature. One-dimensional nanostructures have a high aspect ratio and surface area, and can provide a direct conduction path for electrons. Accordingly, a wide range of ZnO nanostructures [1] such as nanowires (NWs), nanorods (NRs), and nanonails are extensively studied for their applications in various optoelectronic devices, e.g., gas sensors [2], UV photodetectors [3,4], lasers [5,6], electron field emitters [7], solar cells [8-12], and nanogenerators [13].

For most photovoltaic devices, the light is coupled in devices through transparent conductive oxide (TCO) substrate, so tailored well-aligned ZnO nanorod arrays (NRAs) grown on TCO substrate are of particular interest because they can improve the device performance [14]. Previously, ZnO NRAs and NWs on different TCO substrates have been synthesized by various growth methods including chemical bath deposition [8,10,11], electrochemical deposition [9,12,14], and thermal vapor-phase deposition [15,16]. Among these methods, the vapor-phase growth method has many advantages such as excellent crystalline quality of the nanostructures [15], low cost, and simplicity [17]. Generally, ZnO NRs in dye-sensitized solar cells or hybrid solar cells are used to extract the carriers from an organic material and transfer the carriers toward the electrode [15]. Moreover, the density, diameter, length, and crystalline performance of NRs have a significant influence on the efficiency of solar cells [9,15,16]. A larger nanorod diameter will reduce spacing between NRs, which contributes to a reduction in the amount of solar absorber. Longer ZnO NRs do not improve the solar efficiency due to the lower short-circuit current [9]. Therefore, it is important to synthesize ZnO NRAs on TCO substrate with the suitable nanorod diameter, length, and density for their applications in hybrid solar cells. However, there are few reports on the growth and optical properties of ZnO NRAs on a TCO substrate by the vapor-phase deposition [15,16].

In this paper, we focus on the growth and optical properties of ZnO NRAs, which were grown by a solution-free, catalyst-free, vapor-phase synthesis method at a temperature of 600°C. This method can grow ZnO NRAs on Al-doped ZnO (AZO) films, and the performance of AZO does not degrade after the growth of NRAs. AZO has the advantage of being indium free and can be produced on a large scale. The effect of growth duration on the morphology and optical properties of NRAs has been investigated.

## Methods

AZO films were deposited on quartz substrates using a radio-frequency (RF) magnetron sputtering system at room temperature. The quartz substrates, 0.5 mm thick, 2.5 cm × 2.5 cm, were cleaned in acetone and ethanol several times before deposition. The target, 60-mm diameter, was a commercial ZnO and Al_2_O_3_ mixture (97:3 wt.%) of ≥99.99% purity. The sputtering was performed in an Ar atmosphere with a target-to-substrate distance of 5 cm. The base pressure in the chamber was 4.0 × 10^−4^ Pa. The Ar flux determined using a mass flow-controlled regulator was maintained at 50.0 sccm, and the sputtering pressure was 0.5 Pa. The RF power was 300 W, and deposition time was typically 10 min. A typical sheet resistance of AZO film, about 480 nm thick, was about 60 Ω/sq.

ZnO NRAs were grown by a vapor-phase method in a horizontal tube furnace [18]. The substrates, polycrystalline AZO films on quartz substrates, were cleaned in acetone and ethanol before the NRA growth. Commercial zinc (99.99% purity) powder in a ceramic boat was used as the zinc vapor source. The ceramic boat and AZO substrate were placed in a long quartz tube, and the quartz tube was then put into the furnace. An AZO substrate was placed 5 cm downstream from the sources at the heat center of the furnace. After evacuating the system to a base pressure of 12 Pa, the furnace temperature was ramped to 600°C at 20°C min^−1^. A 100-sccm Ar and 10-sccm oxygen mixed gas was introduced into the furnace only when the maximum temperature was reached. The growth pressure was 110 Pa. The temperature was kept at 600°C for several minutes, and then the furnace was cooled down to room temperature. Changing the growth duration, several samples had been synthesized. For simplicity, the samples with growth durations of 3, 6, 8, 9, and 12 min were defined as samples S1, S2, S3, S4, and S5, respectively.

Morphological and structural properties of the grown nanostructures were analyzed using a JSM-7500LV scanning electron microscope (SEM) and a JEM-2010 high-resolution transmission electron microscope (TEM) (JEOL Ltd., Akishima-shi, Japan). For the latter, the samples were prepared by mechanically scraping NRs from the substrate, dispersing them in ethanol, and depositing a drop of the dispersion on a circular copper grid covered by a thin holey carbon film. The crystal structure and orientation were investigated using an X-ray diffractometer (XRD; Y-2000, Rigaku Corporation, Shibuya-ku, Japan) with monochromated Cu Kα irradiation (*λ* = 1.5418 Å). The surface morphology of the AZO film was observed using an atomic force microscope (AFM; CSPM 4000, Benyuan Co. Ltd., Guandong, China) under ambient conditions. The sheet resistance was measured by the van der Pauw method [19].

Room-temperature photoluminescence (PL) spectra of the samples were obtained on a Fluorolog 3–22 fluorescence spectrophotometer (Horiba Ltd., Kyoto, Japan) using a Xe lamp with an excitation wavelength of 325 nm. The total transmittance and diffuse transmittance of the samples were measured using a double-beam spectrophotometer (PerkinElmer Lambda 950, Waltham, MA, USA) equipped with an integrating sphere. In the measurement, the light propagation path was air/quartz/AZO/air or air/quartz/AZO/NRAs/air, and the reflection at the quartz/air interface was not removed.

## Results and discussion

**Figure 1:**
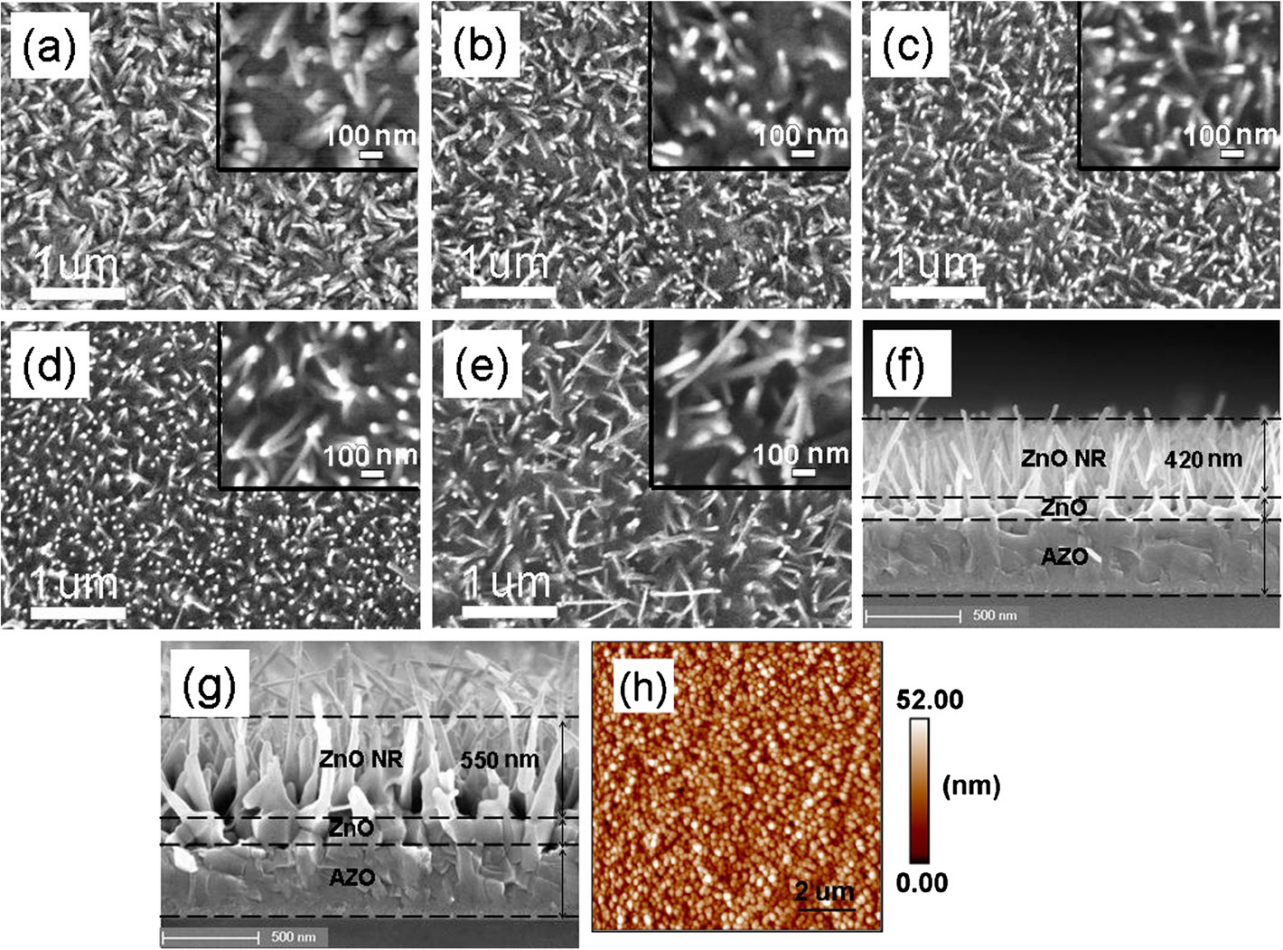
**SEM images of ZnO NRs grown with different durations and AFM surface image of AZO film.** (**a** to **e**) Top-view and (**f**,**g**) cross-sectional SEM images of ZnO NRs grown with different durations: (**a**) S1 - 3 min, (**b**,**f**) S2 - 6 min, (**c**) S3 - 8 min, (**d**) S4 - 9 min, and (**e**,**g**) S5 - 12 min; insets are the high-magnification images of the corresponding samples. (**h**) AFM surface image of AZO film.

The top-view SEM images of samples S1 to S5 are shown in Figures 1a,b,c,d,e, respectively, and the insets are the high-magnification images of the corresponding samples. Figure 1f,g presents the cross-sectional SEM images of samples S2 and S5, respectively. The ZnO NR growth mechanism is the catalyst-free vapor-solid growth due to the absence of metal catalysts on NR tips [20]. Moreover, Figure 1f,g clearly indicates a ZnO buffer layer between NRAs and AZO film, which is used as a seed layer [21]. The density and average NR dimensions of samples S1 to S4 are tabulated in Table 1. Sample S1 has a relatively low NR density, and its NR lengths are between 200 and 300 nm. As the growth duration increases to 8 min, sample S3 has a NR density of 75 μm^−2^, an average NR diameter of 26 nm, and an average length of 500 nm, indicating that the density, length, and aspect ratio of NR increase with the increase of growth duration. The average NR diameter, however, does not obviously change. Moreover, as shown in Figure 1d, the phenomenon of two or three NRs self-attracting in sample S4 with 9-min growth duration can be seen clearly. NRs in sample S5 are out of order because more NRs touch each other and the new NRs grow at NR self-attraction positions. The newly grown NRs are more disordered, and some NRs are almost parallel to the substrate as presented in Figure 1e. As a result, the density and length of the NRs on sample S5 are not calculated in Table 1.

**Table 1:** Density and average NR dimensions (diameter, length, and aspect ratio) of the samples

**Sample**	**Density (per μm**^**2**^**)**	**Average NR diameter 2*r *(nm)**	**Average NR length *L *(nm)**	**Aspect ratio *L*/*r***
S1	40 ± 8	28 ± 7	250 ± 50	17.8
S2	61 ± 6	25 ± 6	420 ± 40	33.6
S3	75 ± 2	26 ± 4	500 ± 20	38.5
S4	82 ± 2	28 ± 4	550 ± 20	39.3

In previous research reports, it was found that the characteristic of ZnO NWs strongly depends on the crystallinity, type, and surface roughness of the growth substrate [20]. The crystallinity, surface roughness, and thickness of the ZnO seed layer also have an important influence on ZnO NR growth [21]. We speculate that two main reasons contribute to the not well vertically aligned NRAs in our samples. First is that the AZO film was deposited on the amorphous quartz substrate, which results in a polycrystalline AZO film as discussed below. Figure 1h is a typical AFM surface image of an AZO film. AFM results indicate that the root-mean-square surface roughness and the average surface particle size are 10.2 and 140 nm, respectively. The second reason, therefore, is that the polycrystalline AZO film deposited by RF sputtering has large surface roughness and surface particle size.

In a hybrid solar cell, ZnO NRs play the roles to extract carriers from the absorber and provide a fast and direct path for these carriers. The efficiency of a solar cell strongly relies on the crystallinity, density, diameter, and length of ZnO NR [9,15]. Conradt et al. [15] have reported that short NRs in the range of 100 to 500 nm are of particular interest for hybrid solar cells. A smaller NR diameter will enhance the spacing between NRs and increase the solar absorber amount and the efficiency of a solar cell [9]. NR in sample S3 has a suitable length about 500 nm and a small diameter about 26 nm. Accordingly, we suggest that sample S3 is interesting for application in hybrid solar cells.

**Figure 2:**
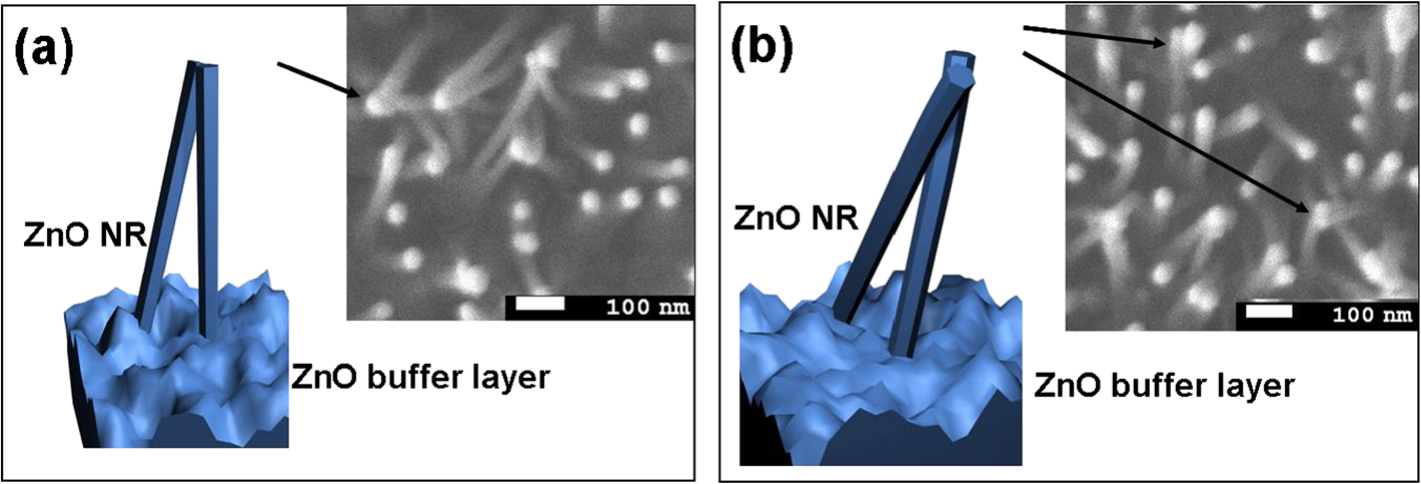
**Schematic diagrams of two possible NR self-attraction models.** (**a**) The tips of two NRs touch each other, (**b**) two NRs touch each other at the crossed position. Insets are top-view images of sample S4.

Most NRs in sample S4 are well aligned, as shown in Figure 1d. However, the phenomenon of two or three NRs self-attracting can be seen obviously in the inset of Figure 1d. Han et al. [22] and Wang et al. [23] had reported self-attraction among aligned ZnO NRs under an electron beam, while Liu et al. [24] have observed the self-attraction of ZnO NWs after the second-time growth. In our samples, NRs with a relatively small diameter are slightly oblique and easily bent, which results in NR self-attraction, given that the NRs are long enough. According to the experimental observation, we propose two possible NR self-attraction models, as presented in Figure 2. The insets in Figure 2 are top-view images of sample S4, and the arrows in the insets denote the examples of the self-attraction models. In the first case, in Figure 2a, NRs randomly grow and are slightly tilted, so the tips of two NRs may just touch each other when the NRs are long enough. In the second case, a NR body may slightly bend due to the oblique growth, which causes the side surfaces to be either positively or negatively charged because of the piezoelectric properties of ZnO NRs [13,24]. As a result, as indicated in Figure 2b, when two bending NRs cross, the opposite charges will lead to the attraction at the crossed position due to the large electrostatic force.

**Figure 3:**
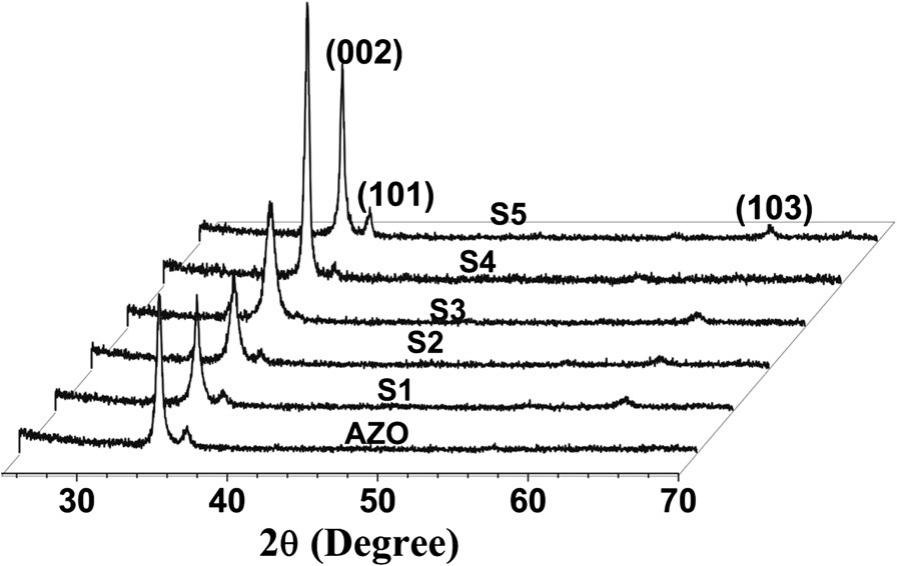
XRD patterns of AZO film and samples S1 to S5.

Figure 3 presents XRD patterns of an AZO film along with the samples. It clearly shows that the AZO film has a preferential orientation along the [001] axis, which is orthogonal to the substrate surface. Additionally, a weak (101) peak indicates that the AZO film is a polycrystalline structure. ZnO NRs grow coherently with the bottom AZO film, maintaining the preferential orientation of the [001] axis. For samples S1 to S4, the intensity of the (002) peak enhances with the increase of growth duration, suggesting that sample S4 has better crystallinity. The reduction of the (002) peak intensity for sample S5 is because the NRs are disordered and have more defects after the new NRs grow at NR self-attraction positions.

**Figure 4:**
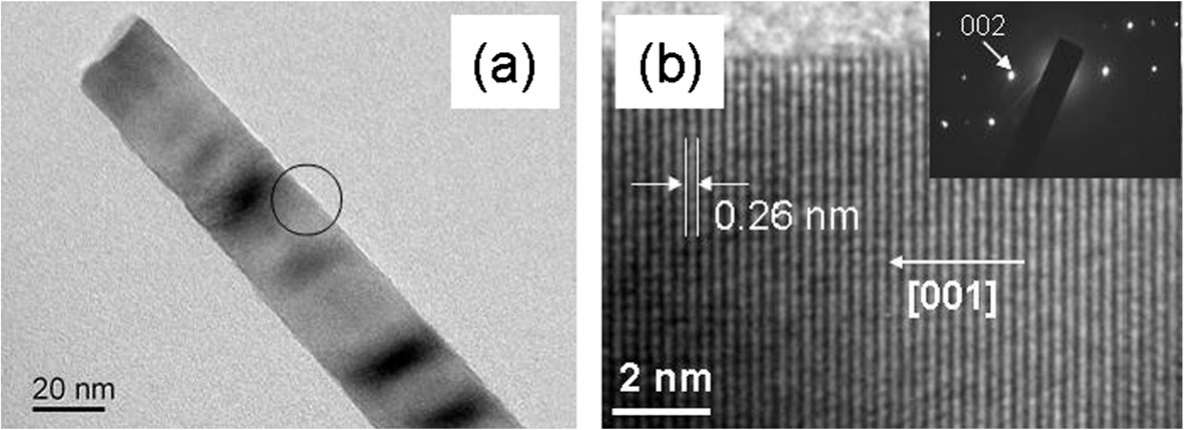
**TEM images of a ZnO NR in sample S3.** (**a**) TEM image of a ZnO NR in sample S3, (**b**) HRTEM image taken at the circle position in (**a**), inset is the corresponding selected-area electron diffraction pattern.

In order to cross-check the crystalline quality of the NRs, a TEM image of a ZnO NR is shown in Figure 4a and clearly indicates the absence of metal catalysts on the ending. In a high-resolution TEM image, Figure 4b, continuous crystal planes can be seen, which are perpendicular to the growth direction and exhibit an interplanar distance of 0.26 nm. The inset in Figure 4b presents the selected-area electron diffraction pattern from this NR, which suggests that NR is the single-crystal ZnO with wurtzite structure.

**Figure 5:**
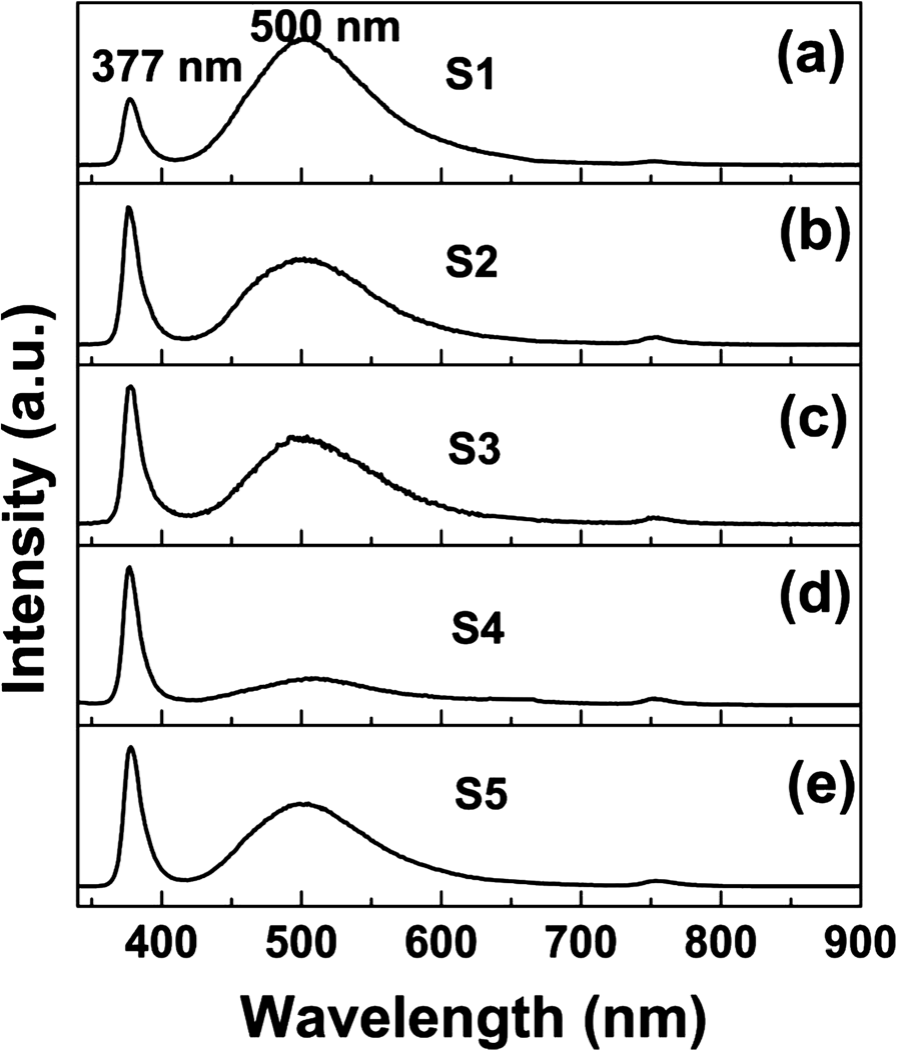
**PL spectra of samples.** (**a**) to (**e**) are samples S1 to S5.

Room-temperature PL properties of ZnO NRAs of samples S1 to S5 are shown in Figure 5. There are two emission peaks in the PL spectra. One peak located at about 377 nm is the near-band-edge emission or UV emission, and the other green band peak at about 500 nm is the deep-level emission [3]. The relative PL peak intensity ratio (*R* = *I*_UV_ / *I*_DLE_) is defined as a figure of merit. *R* is 0.5, 1.6, 1.6, 5.1, and 1.7 for samples S1, S2, S3, S4, and S5, respectively. Comparing samples S1 to S4, it is found that *R* enhances with the increase of growth duration, which is due to the decrease of oxygen vacancies [18]. Sample S1 has the strongest deep-level emission because it has the most oxygen vacancies and the shortest oxidation time. Although sample S5, however, has the longest growth duration, its deep-level-emission is relatively strong. This is because the new NRs grown at NR self-attraction positions have worse crystallinity, as shown in Figure 3, shorter growth duration, and more oxygen vacancies.

**Figure 6:**
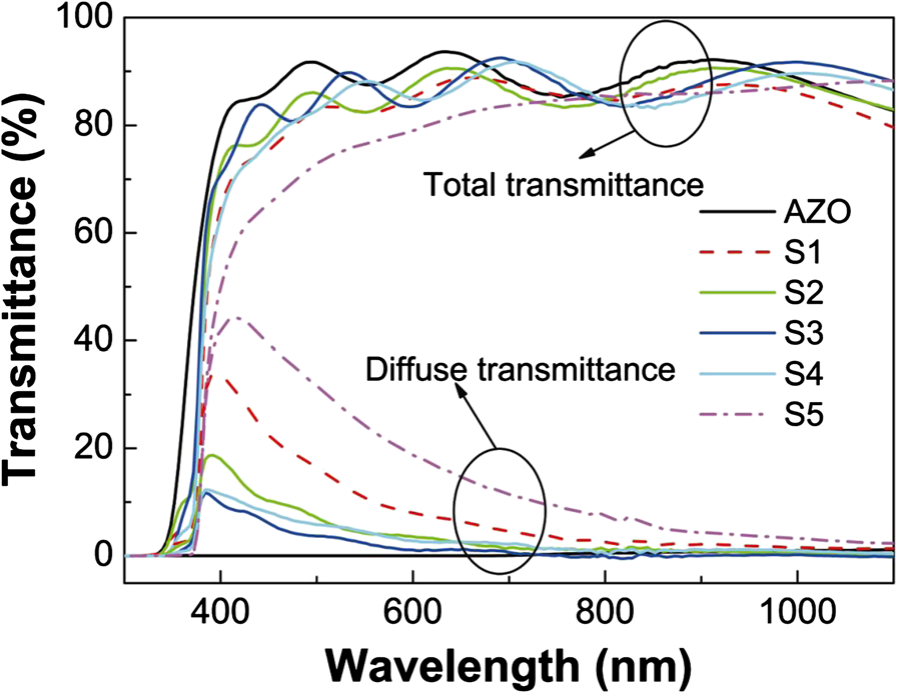
Total and diffuse transmittances of samples S1 to S5.

Semiconductor nanostructures offer a powerful tool to efficiently manage the light in photovoltaic devices, and the morphology of NWs or NRs has a significant effect on their transmittance and reflectance [14,25,26]. The total and diffuse transmittance spectra of the samples were measured, and the results are presented in Figure 6. The average total transmittance (ATT) and average diffuse transmittance (ADT) in the wavelength range of 400 to 1,100 nm are shown in Table 2. ATT and ADT of the AZO film are 88.6% and 0.4%, respectively, indicating that the AZO film has good transparence. ATTs of samples S1 to S5 are higher than 80%. The highest diffuse transmittance of sample S5 is 44% at 416-nm wavelength. The diffuse transmittance decreases and total transmittance increases with increasing wavelength when the wavelength is larger than 416 nm. Sample S3 has the highest ATT and the lowest ADT because its NRs are more vertically aligned, as shown in Figure 1. NRs in sample S5 are disordered (Figure 1e) and have more oxygen vacancies, as discussed in the PL spectra, which results in the lowest ATT and the highest ADT of sample S5. For sample S1, although the NRs are relatively ordered, the low NR density and short NR length (Figure 1a) strongly enhance the optical surface scattering [27]. As a result, sample S1 has a large diffuse transmittance.

**Table 2:** ATT, ADT, and SR of the AZO film and samples

**Sample**	**AZO**	**S1**	**S2**	**S3**	**S4**	**S5**
ATT (%)	88.6	84.0	85.7	87.0	85.5	81.0
ADT (%)	0.4	7.3	3.2	1.5	2.8	14.2
SR (Ω/sq)	60	17	33	48	44	36

An AZO film must have a low resistance for use as a transparent conductive electrode in optoelectronic devices [16]. The electrical properties of an AZO film may be changed after thermal treatment at high temperature, and especially our NR growth temperature is 600°C. So, the sheet resistance (SR) of the sample was measured. The NRs at electrode positions were removed to enable good contact of the electrodes before the resistance measurement, and the results are shown in Table 2. All the sheet resistances of the samples are lower than that of the AZO film (60 Ω/sq), indicating that the electrical performance of the AZO film does not degenerate after the NR growth. We speculate that there are two mechanisms that induce the reduction of the sheet resistances. One is that the resistance of the AZO film after the thermal treatment declines, which had been confirmed experimentally [16,28]. The other is, as indicated in Figure 1f,g, the result of a ZnO buffer layer between NRAs and AZO film after NR growth. ZnO is naturally an n-type semiconductor due to the presence of intrinsic defects such as oxygen vacancies and zinc interstitials [29]. The resistance of a ZnO film will decline as the oxygen vacancies increase because each oxygen vacancy can generate two conductive electrons. The NRAs and ZnO buffer layer in sample S1 have the most oxygen vacancies, as confirmed by PL measurement, so it has the lowest sheet resistance (17 Ω/sq).

## Conclusions

A solution-free, catalyst-free, vapor-phase growth method was used to synthesize ZnO nanorod arrays on AZO films, which were deposited on quartz substrates by RF magnetron sputtering. The sheet resistance of the sample declines after ZnO NRA growth at 600°C. TEM results show that the NRs are the single-crystal ZnO with wurtzite structure. As the growth duration increases from 3 to 8 min, the oxygen vacancies and diffuse transmittance of the samples decrease, while the crystallinity, aspect ratio, near-band-edge emission, and total transmittance enhance. ZnO NR self-attraction in the sample with 9-min growth duration has been observed, and two possible NR self-attraction models are proposed. NRs in the sample with 12-min growth duration are disordered, which has the largest diffuse transmittance and a relatively strong deep-level emission. The sample with 8-min growth duration has a density about 75 μm^−2^, diameter about 26 nm, and length about 500 nm, which can be used in a hybrid solar cell.

## Competing interests

The authors declare that they have no competing interests.

## Authors’ contributions

SL grew the nanorods and performed the tests on the samples. HH participated in the analysis of TEM results. WZ deposited the Al-doped ZnO films. YQ participated in the test of the samples. FL designed the study and drafted the manuscript. All authors read and approved the final manuscript.
